# Phosphorylation of *Mycobacterium tuberculosis* Ser/Thr Phosphatase by PknA and PknB

**DOI:** 10.1371/journal.pone.0017871

**Published:** 2011-03-09

**Authors:** Andaleeb Sajid, Gunjan Arora, Meetu Gupta, Sandeep Upadhyay, Vinay K. Nandicoori, Yogendra Singh

**Affiliations:** 1 Institute of Genomics and Integrative Biology (CSIR), Delhi, India; 2 National Institute of Immunology, Aruna Asaf Ali Marg, New Delhi, India; Tulane University, United States of America

## Abstract

**Background:**

The integrated functions of 11 Ser/Thr protein kinases (STPKs) and one phosphatase manipulate the phosphorylation levels of critical proteins in *Mycobacterium tuberculosis*. In this study, we show that the lone Ser/Thr phosphatase (PstP) is regulated through phosphorylation by STPKs.

**Principal Findings:**

PstP is phosphorylated by PknA and PknB and phosphorylation is influenced by the presence of Zn^2+^-ions and inorganic phosphate (Pi). PstP is differentially phosphorylated on the cytosolic domain with Thr^137^, Thr^141^, Thr^174^ and Thr^290^ being the target residues of PknB while Thr^137^ and Thr^174^ are phosphorylated by PknA. The Mn^2+^-ion binding residues Asp^38^ and Asp^229^ are critical for the optimal activity of PstP and substitution of these residues affects its phosphorylation status. Native PstP and its phosphatase deficient mutant PstP_c_^D38G^ are phosphorylated by PknA and PknB in *E. coli* and addition of Zn^2+^/Pi in the culture conditions affect the phosphorylation level of PstP. Interestingly, the phosphorylated phosphatase is more active than its unphosphorylated equivalent.

**Conclusions and Significance:**

This study establishes the novel mechanisms for regulation of mycobacterial Ser/Thr phosphatase. The results indicate that STPKs and PstP may regulate the signaling through mutually dependent mechanisms. Consequently, PstP phosphorylation may play a critical role in regulating its own activity. Since, the equilibrium between phosphorylated and non-phosphorylated states of mycobacterial proteins is still unexplained, understanding the regulation of PstP may help in deciphering the signal transduction pathways mediated by STPKs and the reversibility of the phenomena.

## Introduction

*Mycobacterium tuberculosis* has an array of proteins to ensure its existence during the course of infection. In order to thrive and maintain its homeostasis, the pathogen continuously influences its surroundings mainly through surface-located sensor proteins. Extracellular signals are communicated through the sensors to the cytosol leading to the appropriate cell responses. Apparently, a large number of pathogens employ reversible phosphorylation of proteins by kinases and phosphatases as a way of transmitting the signals from extracellular milieu which helps in their survival and pathogenicity [1]–[4]. Kinases carry out the phosphorylation by transferring the phosphate moiety on target proteins and phosphatases convert them back to the unphosphorylated state, either by dephosphorylating the substrate or by regulating the activity of kinases.

Apart from the well recognized two component systems targeting His/Asp residues in bacteria, Ser, Thr and Tyr residues are also the major targets for phosphorylation. *M. tuberculosis* is known to have 11 Ser/Thr protein kinases (STPKs PknA-L, except C), one tyrosine kinase (PtkA), one Ser/Thr phosphatase (PstP) and two tyrosine phosphatases (PtpA and PtpB) [5], [6]. Till date a large number of mycobacterial proteins are shown to be regulated through phosphorylation by STPKs [7]–[11]. Some of these substrates are also known to be dephosphorylated by PstP [9], [11]–[17]. PstP is a PP2C phosphatase (PPM family) that strictly requires Mn^2+^-ion for its activity [13]. It is a membrane localized enzyme with intracellular catalytic domain of 237 amino acids joined by a juxtamembrane region to the extracellular domain of 191 residues with a single transmembrane helix [18]. Using multi-wavelength anomalous diffraction studies, Pullen *et* al. determined the structure of the catalytic phosphatase domain of PstP [18]. PstP contains three metal-binding centers in its structure in contrast to two metal centers found in most of the PP2C phosphatases. Using atomic absorption spectroscopy and X-ray analysis, it has been shown that all the bound metal-ions are Mn^2+^. Similarities between Human Ser/Thr phosphatase PP2Cα and the mycobacterial enzyme have been explained on the basis of structural folds, metal binding and conserved residues [18]. Mutational analyses of PP2Cα have depicted the significance of certain conserved amino acid residues [19]. The corresponding residues in PstP are involved in binding to metal-ions and catalysis in addition to managing the binding and release of phosphate moiety. These residues in PP2Cα are critical for its activity [19] and thus, they are hypothesized to be important for PstP also.

The interesting feature of *M. tuberculosis* Ser/Thr signaling molecules is that both the essential STPKs, PknB (Rv0014c) and PknA (Rv0015c) and the only Ser/Thr phosphatase PstP (Rv0018c) are located in the same genomic cluster which is conserved in several mycobacterial species [6], [9], [20]. Transcriptional analysis in earlier studies revealed that PknA, PknB and PstP show similar expression profiles [20] and thus, implicate that strong regulation is required for their own functions as both the classes of enzymes functionally counteract each other. In this study, we show that the activity of PstP is modulated by phosphorylation. This is the first report on the regulation of any bacterial Ser/Thr phosphatase by post-translation modification. PstP was found to be phosphorylated differentially by PknA and PknB, both *in vitro* and in the surrogate host *Escherichia coli*. Additionally, we found that zinc ions (Zn^2+^) and inorganic phosphate (Pi) can inhibit the activity of PstP which in turn affects the phosphorylation status of both the kinases and phosphatase.

## Materials and Methods

### Bacterial strains and growth conditions

*E. coli* DH5α strain (Novagen) was used for cloning and BL21 (DE3) (Stratagene) was used for the expression of recombinant proteins. *E. coli* cells were grown and maintained with constant shaking (220 rpm) at 37°C in LB medium supplemented with 100 µg/ml ampicillin.

### Gene manipulation

The genes coding for PknA_c_ (*rv0015c*, representing the **c**ytosolic region of 1-337aa) and PstP (*rv0018c*, PstP: 1-514aa) were PCR amplified using *M. tuberculosis* H37Rv genomic DNA. Resulting PCR products were digested with corresponding restriction enzymes and ligated into the vectors pProEx-HTc (Invitrogen) and/or pGEX-5X-3 (GE Healthcare Bio-Sciences) previously digested with the same enzymes. *Htc-PknB_c_* and *Htc-PstP_c_* were obtained as described earlier [9]. *pGEX-PknB_c_* was sub-cloned from *Htc-PknB_c_* using standard protocols under the same restriction sites. For cloning in dual-expression vector pETDuet-1 (Novagen), genes coding for PstP_c_ or PstP_c_^D38G^ were inserted in MCS1 having N-terminal His_6_-tag while kinases PknA and PknB (full length) were cloned in MCS2 with N-terminal MBP-tag (Maltose-binding protein tag upstream of the kinase). MBP-alone (without kinase) was taken as control vector having PstP_c_ or PstP_c_^D38G^ in MCS1. The protocols used for cloning in pETDuet-1 have been discussed earlier [21].

Mutagenesis of specific residues was carried out using the QuikChange XL site-directed mutagenesis kit (Stratagene) as per manufacturer's instructions. Mutants of PstP and PstP_c_ were created as R20G, D38G and D229G using *Htc-PstP* and *Htc-PstP_c_* as templates. *Htc-PstP_c_* was utilized for the generation of *Htc-PstP_c_^t5a^* and *Htc-PstP_c_^t141e^*. *Htc-pknB_c_* was employed as template for generation of double mutant *Htc-pknB_c_^t171/173d^*. The details of all the primers and clones are provided in tables 1 and 2, respectively. The integrity of all clones was confirmed by DNA sequencing (TCGA, New Delhi).

**Table 1:** Primers used in the study.

Primer Name	Sequence Details (5′→3′) **
PknB_c_^T171/173D^ FP	CGGCAACAGCGTGGACCAGGACGCAGCAGTGATCG
PknB_c_^T171/173D^ RP	CGATCACTGCTGCGTCCTGGTCCACGCTGTTGCCG
PknA FP	TGATCGAAGCCGGAATTCAGGGGGAACCATGA EcoR1
PknA_c_ RP	AGCACCCCCGCGGCCGCGAGCAGCGCTCACTGACCGGAC Not1
PstP_c_^D38G^ FP	CTATTGGCCCTGGCCGGCGGCATGGGTGGGCAT
PstP_c_^D38G^ RP	ATGCCCACCCATGCCGCCGGCCAGGGCCAATAG
PstP_c_^R20G^ FP	GATCGCGGCTTGGTAGGCGCCAACAACGAAGACTCGGTC
PstP_c_^R20G^ RP	GACCGAGTCTTCGTTGTTGGCGCCTACCAAGCCGCGATC
PstP_c_^D229G^ FP	GGCGGCGGCCCCGGCAACGTCACTGTCGTCGTC
PstP_c_^D229G^ RP	GACGACGACAGTGACGTTGCCGGGGCCGCCGCC
PstP_c_^T5A^ FP	GGAGAGTGGCGCGCGTGGCCCTGGTCCTGCGATAC
PstP_c_^T5A^ RP	GTATCGCAGGACCAGGGCCACGCGCGCCACTCTCC
PstP_c_^T141E^ FP	GACGACACGTTTGTCCAAGCGCTGGTCGACGAAGGCCG
PstP_c_^T141E^ RP	CGGCCTTCGTCGACCAGCGCTTGGACAAACGTGTCGTC
pETDuet-PstP FP	CACC GCGGCCGCTCATATG GCGCGCGTGACCCTGG Not1
pETDuet-PstP_c_ RP	CGGTCACCAGTGCGGCCGCGAATGCTCACCGTCGGCC Not1

**Restriction sites/stop codon/mutated sequences have been underlined.

**Table 2:** Description of the plasmids used in this study.

Plasmid construct	Description	Reference
pProEx-HTc	*E. coli* expression vector containing N-terminal His_6_-tag	Invitrogen
pProEx-HTc-PknB_c_	Expression of His_6_PknB_1-331_ (cytosolic domain)	[9]
pProEx-HTc-PknB_c_^T171/173D^	pProEx-HTc-PknB_c_ with activation loop residues Thr^171^ and Thr^173^ mutated to Asp, phosphomimetic amino acid	This study
pProEx-HTc-PknA_c_	Expression of His_6_PknA_1-337_ (cytosolic domain)	This study
pProEx-HTc-PstP_c_	Expression of His_6_PstP_1-300_ (cytosolic domain)	[9]
pProEx-HTc-PstP_c_^R20G^	pProEx-HTc-PstP_c_ with Arg^20^ mutated to Gly	This study
pProEx-HTc-PstP_c_^D38G^	pProEx-HTc-PstP_c_ with Asp^38^ mutated to Gly	This study
pProEx-HTc-PstP_c_^D229G^	pProEx-HTc-PstP_c_ with Asp^229^ mutated to Gly	This study
pProEx-HTc-PstP_c_^T5A^	pProEx-HTc-PstP_c_ with Thr^5^ mutated to Ala	This study
pProEx-HTc-PstP_c_^T141E^	pProEx-HTc-PstP_c_ with Thr^141^ mutated to Glu	This study
pGEX-5X-3	*E. coli* expression vector containing N-terminal Glutathione S-Transferase tag	GE Healthcare
pGEX-5X-3-PknA_c_	Expression of GST-PknA_1-337_ (cytosolic domain)	This study
pGEX-5X-3-PknB_c_	Expression of GST-PknB_1-331_ (cytosolic domain)	This study
pETDuet1	*E. coli* dual expression vector containing N-terminal His_6_-tag in MCS1 and C-terminal S-tag in MCS2	Novagen
pETDuet1-PstP_c_^D38G^/MBP	Expression of His_6_-PstP_c_^D38G^ in MCS1 with Myelin basic protein (MBP) in MCS2	This study, [21]
pETDuet1-PstP_c_^D38G^/MBP-PknA	Expression of His_6_-PstP_c_^D38G^ in MCS1 with MBP-tagged PknA in MCS2	This study, [21]
pETDuet1-PstP_c_^D38G^/MBP-PknB	Expression of His_6_-PstP_c_^D38G^ in MCS1 with MBP-tagged PknB in MCS2	This study, [21]

### Protein expression and purification

Proteins were expressed and purified from *E. coli* as described previously [9]. The purified proteins were assessed by SDS-PAGE and concentrations were estimated by Bradford assay (Bio-Rad).

### *In vitro* kinase assays and phosphoamino acid analysis

*In vitro* phosphorylation of PstP_c_ or its mutants (0.5–3 µg) by PknA_c_ (0.5–1 µg) or PknB_c_ (1–3 µg) was carried out in kinase buffer (20 mM PIPES [pH 7.2], 5 mM MnCl_2_, 5 mM MgCl_2_) containing 2 µCi [γ-^32^P]ATP (BRIT, Hyderabad, India) followed by incubation at 25°C for 20 min. Reactions were terminated by 5X SDS sample buffer followed by boiling at 100°C for 5 min. Proteins were separated by 12% SDS-PAGE and analyzed by PhosphorImager (FLA 2000, Fuji). Zn^2+^ and Pi were added to the kinase assay reactions as per requirement of the assay. For the visualization of phosphorylation signal on cleaved proteins, removal of recombinant tags was achieved by addition of TEV protease (for His_6_-tagged PstP/PstP_c_ and their mutants) in TEV buffer (Tris-Cl [pH 8.5], 5 mM EDTA, 300 mM NaCl and 1 mM DTT) after the kinase reaction followed by an additional incubation for 2 hr at 20°C. For phosphoamino acid analysis, PstP_c_^D38G^ was phosphorylated by PknB_c_ and PknA_c_ and cleaved with TEV protease as mentioned above, separated by SDS-PAGE and electroblotted onto Immobilon PVDF membrane (Millipore). Phosphoamino acid analysis by two-dimensional thin layer electrophoresis was performed as described earlier [9], [22].

### *In vitro* dephosphorylation and *p*-nitrophenol phosphate (*p*NPP) hydrolysis assays

PknB_c_ and PknA_c_ were autophosphorylated by *in vitro* kinase assays using [γ-^32^P]ATP. 1 µg of purified PstP_c_/PstP_c_^D38G^/PstP_c_^R20G^/PstP_c_^D229G^ were added in four sets of reactions and incubated at 25°C for increasing time points up to 30 min to measure the dephosphorylation potential of PstP_c_ and its mutants. For auto-dephosphorylation assays, PknB_c_ and PknB_c_^T171/173D^ (2 µg each) were autophosphorylated by *in vitro* kinase assays and exposed to dephosphorylation by PstP_c_ and PstP_c_^D38G^ (1 µg). Reactions were stopped by adding 5X SDS sample buffer and boiled for 5 min at 100°C. The samples were separated by 12% SDS-PAGE and phosphorylated bands were observed and analysed by PhosphorImager.

*p*NPP hydrolysis assay was performed as a measure of phosphatase activity. PstP_c_ was added to a reaction mixture containing phosphatase assay buffer (50 mM Tris pH 8.0, 5 mM DTT, 4 mM MnCl_2_) and 10 mM *p*NPP in a 96-well plate and incubated at 37°C for indicated time points and absorbance was read at 405 nm (Microplate reader, Bio-Rad). To assay the relative activity of PstP_c_ and its phosphatase-deficient variants, increasing concentrations of enzymes were added to the reaction mix and processed as above. Alkaline phosphatase (Roche) and PknB_c_ were taken as positive and negative controls, respectively, for the *p*NPP hydrolysis assays. Variations of PstP_c_ activity by addition of Zn^2+^ and Pi was assessed by adding ZnCl_2_ or sodium phosphate [pH 7.2] to the reaction mixture as above, to achieve the indicated final concentrations. pETDuet-1 purified PstP_c_ and PstP_c_^D38G^, co-expressed with or without kinases, were employed for *p*NPP-assays to measure the effect of phosphorylation on their activities.

### Metabolic labeling in *E. coli*

The procedure described by Kumar *et* al. was followed for metabolic labeling [23]. *E. coli* (BL21-DE3) transformants harbouring either *pETDuet-PstP_c_/PstP_c_^D38G^-mbp* or *pETDuet-PstP_c_/PstP_c_^D38G^-mbpPknA* or *pETDuet-PstP_c_/PstP_c_^D38G^-mbpPknB* were grown in 5 ml LB medium containing 100 µg/ml ampicillin to an O.D_600_ of ∼0.6. The cells were induced with 1 mM IPTG and further grown for 4 hr at 16°C. Cultures were harvested, washed with 5 ml of M9 medium [pH 7.0] without phosphate salts (for 1 L: NH_4_Cl-1 g, NaCl-0.5 g, 20% Glucose-10 ml, MgSO_4_.7H_2_O-1 ml, Thiamine-HCl-1 ml, CaCl_2_-1 ml). The cells were resuspended in 2 ml of M9 media supplemented with 1 mCi of [^32^P]orthophosphoric acid (BRIT, Hyderabad, India), 100 µg/ml ampicillin and 1 mM IPTG and further grown at 16°C for 4 hr. Under specific conditions, Sodium phosphate [pH 7.2] (2 mM) or ZnCl_2_ (4 mM) were added to M9 media and subsequent processing steps of metabolic labeling. The cells were harvested and lysed by sonication in the lysis buffer containing phosphate-buffered saline, 5% glycerol and protease inhibitor cocktail. The cell lysate was clarified and the lysates containing His_6_-fusion protein were incubated with lysis buffer equilibrated Ni^2+^-NTA affinity beads for 2 hr at 4°C. The beads were then thoroughly washed with lysis buffer containing 20 mM imidazole and resuspended in 5X SDS sample buffer followed by boiling for 15 min. The samples were resolved on SDS-PAGE followed by autoradiography.

### Identification of phosphorylation sites in PstP_c_^D38G^

PknB_c_ and PknA_c_ were employed for *in vitro* kinase assay using 50 µM cold ATP and PstP_c_^D38G^. The samples were run on 12% SDS-PAGE, stained with Coomassie Brilliant Blue and de-stained. Bands corresponding to PstP_c_^D38G^ were excised from the gel and washed with MilliQ water. The samples were processed for identification of phosphorylation sites by using Thermo-Finnagen LTQ electrospray instrument (Proteomics Core Facility, Children's Hospital, Boston). The detailed protocol of sample processing for identification of phosphorylation sites has been provided in File S1.

### Generation of polyclonal antibodies for PstP_c_ in rabbit and immunoblotting

Polyclonal antibodies against PstP_c_ were generated in rabbit. To confirm the presence of PstP_c_/PstP_c_^D38G^ in Ni^2+^-NTA pulled-out proteins after metabolic labeling by western blot analysis, the samples were resolved by SDS-PAGE along with positive (purified PstP_c_) and negative controls (GST-PknB_c_) and transferred onto nitrocellulose membrane (Bio-Rad). Standard procedure for immunoblotting was followed [9], [11]. The blots were developed using SuperSignal^R^ West Pico Chemiluminescent Substrate kit (Pierce Protein Research Products) according to manufacturer's instructions.

## Results

### Identification of the residues critical for the activity of PstP

**Figure 1:**
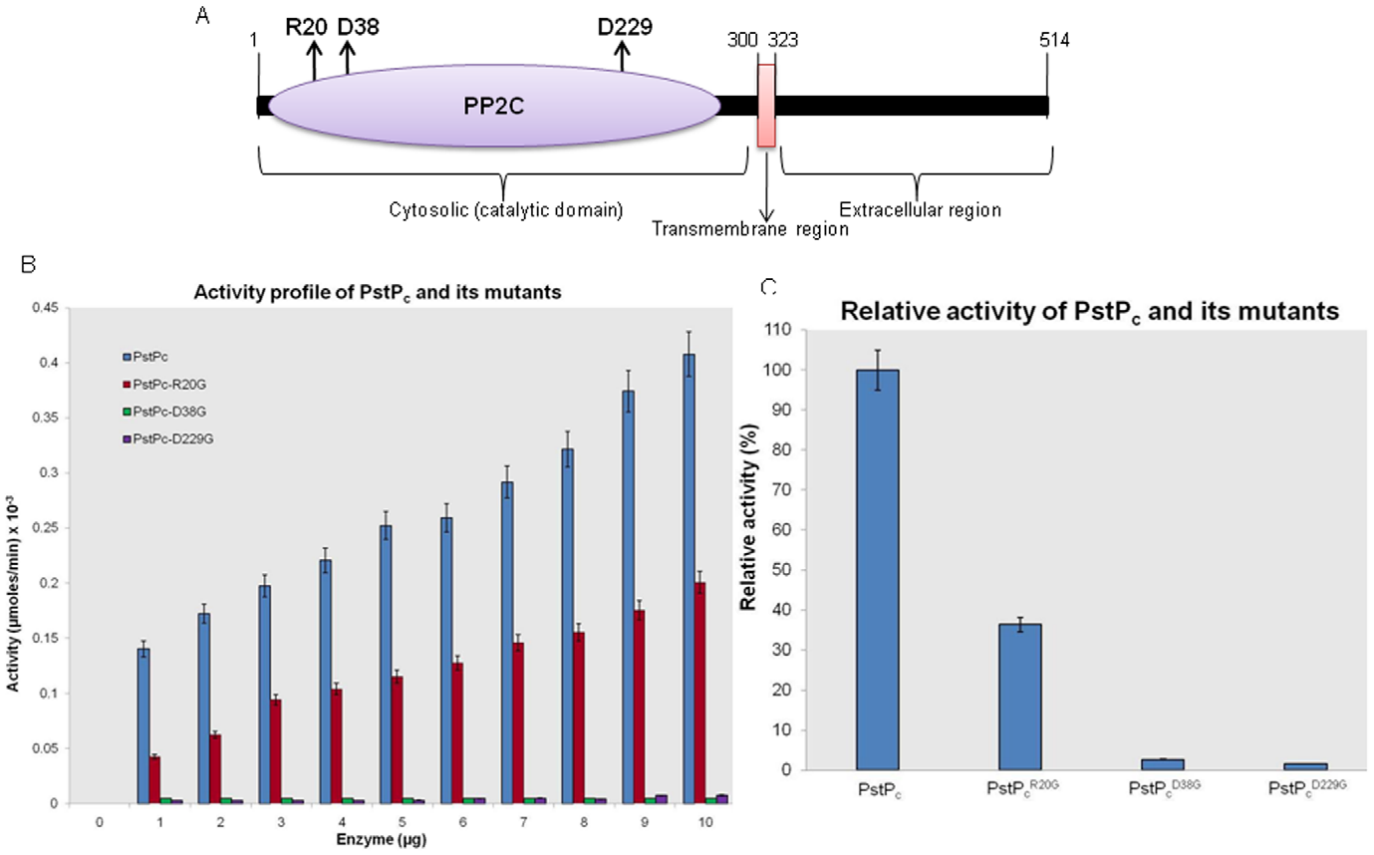
Critical residues of PstP. (**A**) Schematic representation of PstP with critical residues (Arg^20^, Asp^38^ and Asp^229^) being highlighted with upward arrows. (**B**) Activity profiles of PstP_c_ and its mutants: Activity assays were performed by *p*NPP-hydrolysis mediated by PstP_c_, PstP_c_^R20G^, PstP_c_^D38G^ and PstP_c_^D229G^. Increasing concentrations of proteins were taken with constant substrate concentration (10 mM *p*NPP) and incubated at 37°C for 30 mins. As shown in the graph, the mutants had lost phosphatase activity to different extents. Activity is calculated as a measure of µmoles of *p*NPP hydrolyzed per min. at a given enzyme concentration. (**C**) The relative activity of all the phosphatase variants (5 µg each, 30 min.) showed that PstP_c_^D38G^ and PstP_c_^D229G^ had lost >90% of activity while PstP_c_^R20G^ lost ∼60% of the activity as compared to PstP_c_. The error bars indicate the SD of three individual experiments.

On the basis of structural data available for PstP and alignment with the residues important for Human PP2Cα activity [18], PstP_c_ mutants were generated using site-directed mutagenesis. These residues include the Mn^2+^-ion binding sites-Asp^38^ and Asp^229^ and phosphate (Pi) binding residue-Arg^20^ (Figure 1A). In the resulting mutants, these sites were converted to Glycine (PstP_c_^D38G^, PstP_c_^D229G^ and PstP_c_^R20G^). The activity of these mutants was compared using chromogenic substrate *p*NPP. To confirm the authenticity of the assay, increasing concentrations of alkaline phosphatase were utilized as a positive control while PknB_c_ was used as negative control (Figure S1). The *p*NPP assay with increasing amounts of PstP_c_-mutants showed that the mutation of Asp^38^ and Asp^229^ to Gly resulted in >90% loss of the dephosphorylation activity of PstP_c_, while the PstP_c_^R20G^ mutant lost about 60% of its activity (Figure 1B and 1C). Thus, Arg^20^, Asp^38^ and Asp^229^ were identified as the residues required for optimum activity of PstP. To confirm that the loss in activity was specifically due to mutagenesis of Asp^38^, Asp^229^ and Arg^20^, irrelevant residues (Thr^5^ and Thr^141^) in PstP_c_ were mutagenized to generate PstP_c_^T5A^ and PstP_c_^T141E^. The relative activities of these mutants were compared with the native enzyme through *p*NPP-assay (Figure S2). There were no significant changes observed in the mutants in comparison to PstP_c_, thus reinforcing the importance of Arg^20^, Asp^38^ and Asp^229^ residues.

### Phosphatase activity of PstP_c_ and its mutants

**Figure 2:**
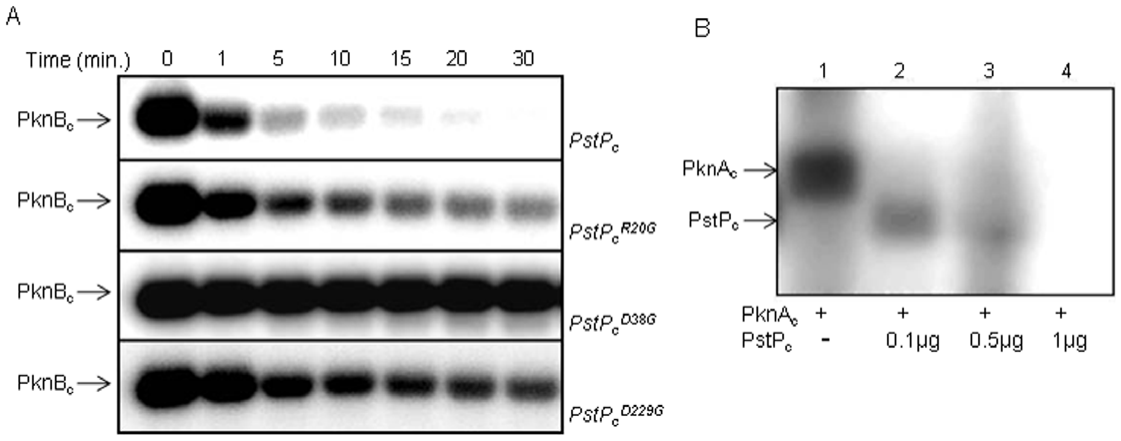
Dephosphorylation by PstP_c_ and its mutants. (**A**) Autoradiogram showing autophosphorylated PknB_c_, exposed to dephosphorylation by PstP_c_, PstP_c_^R20G^, PstP_c_^D38G^ and PstP_c_^D229G^. Time-dependent dephosphorylation was performed with 1 µg of phosphatase after carrying out autophosphorylation of PknB_c_ (2 µg) in an *in vitro* kinase assay. Noticeably, PstP_c_^D38G^ was observed to be phosphorylated with increasing time points (3^rd^ panel from the top). (**B**) Autoradiogram showing phosphorylation of PstP_c_ by PknA_c_ (1 µg). Increasing concentrations of PstP_c_ were used to measure the extent of dephosphorylation. Unexpectedly, the phosphatase itself got phosphorylated at higher kinase to phosphatase ratio, though kinase was completely dephosphorylated. No phosphorylation was observed at higher PstP_c_ concentrations.

The dephosphorylation potential of PstP_c_ and its mutants was also assessed by their ability to dephosphorylate PknB_c_ in a time-dependent dephosphorylation (Figure 2A) and *p*NPP hydrolysis assays (Figure S3). PstP_c_^R20G^ dephosphorylated the autophosphorylated PknB_c_ to some extent, whereas substantial loss of phosphatase activity was observed with PstP_c_^D38G^ and PstP_c_^D229G^ (Figure 2A). The activity of PstP_c_^D229G^ was relatively higher than that of PstP_c_^D38G^ as opposed to the observation in *p*NPP-assays (Figures 1C and S3). Similar observations have been reported earlier where the activity of an enzyme, specifically Ser/Thr phosphatases, is shown to be dependent on the nature of substrate [24]–[26]. *p*NPP is an artificial substrate while PknB is a natural substrate of PstP, which may be recognized and subsequently dephosphorylated more optimally. Additionally, in this case, the activity of the phosphatase also depends on the activity of PknB, as discussed in later sections. The assays were also performed using autophosphorylated PknA_c_ which showed similar results (data not shown). Surprisingly, in this assay, additional phosphorylated bands corresponding to the size of PstP_c_^D38G^ were observed when incubated with kinase for longer time. No such bands were observed with PstP_c_, PstP_c_^R20G^ and PstP_c_^D229G^ at the given concentrations.

To further assess this observation, PknA_c_ or PknB_c_ were incubated with increasing concentrations of PstP_c_. Interestingly, PstP_c_ was phosphorylated by PknA_c_ at higher kinase to phosphatase ratio (Figure 2B). An increase in the concentration of PstP_c_ resulted in complete dephosphorylation of both the proteins. This serendipitous observation intrigued us to explore whether PstP is a target of Ser/Thr protein kinases. Due to strong dephosphorylation activity of PstP, it was difficult to achieve the phosphotransfer on native phosphatase. Therefore, further studies were carried out with the mutants of PstP that were deficient in phosphatase activity.

### Phosphorylation of PstP_c_^D38G^, PstP_c_^D229G^ and PstP_c_^R20G^

**Figure 3:**
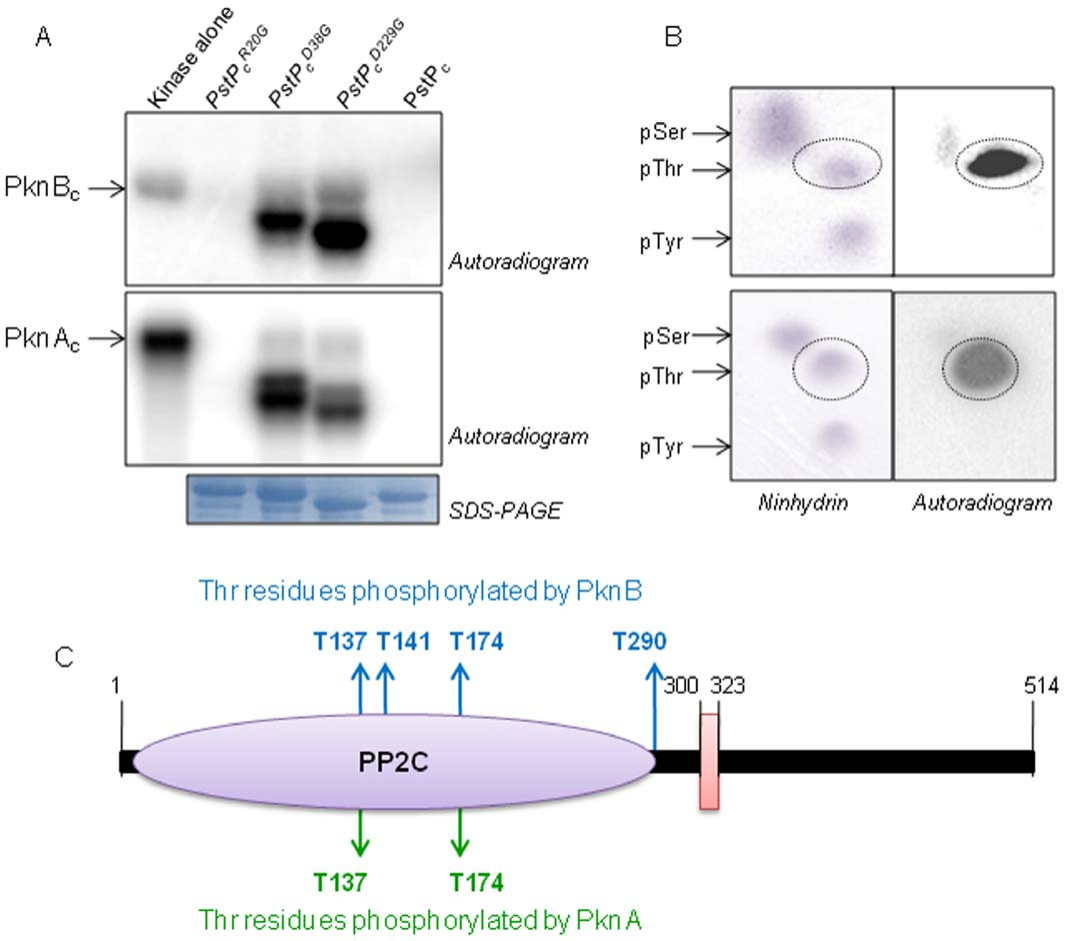
Phosphorylation of PstP_c_ and its mutants by PknA and PknB. (**A**) Phosphorylation of PstP_c_ and its mutants (3 µg each) by 2 µg PknB_c_ (upper panel) and 0.5 µg PknA_c_ (middle panel). PstP_c_^D38G^ and PstP_c_^D229G^ were efficiently phosphorylated by both the kinases due to loss of phosphatase activity. Phosphorylation on PstP_c_^R20G^ mutant was low due to its partial phosphatase activity. The corresponding SDS-PAGE is shown (lowest panel) as a loading control. (**B**) Phosphoamino acid analysis by 2D-TLE illustrates that both PknA_c_ (upper panel) and PknB_c_ (lower panel) phosphorylates PstP_c_^D38G^ on Thr residues. (**C**) Sites of phosphorylation of PknB_c_ (blue) and PknA_c_ (green) in PstP_c_^D38G^ were identified by mass spectrometric analysis. PknB_c_ phosphorylates PstP_c_^D38G^ majorly on four Thr residues-Thr^137^, Thr^141^, Thr^174^ and Thr^290^ while two Thr residues were phosphorylated by PknA_c_-Thr^137^ and Thr^174^.

After identification of the residues critical for PstP_c_ activity and measuring the activity of corresponding mutants, the phosphorylation status of PstP_c_ mutants was studied. PknA and PknB were employed for the phosphorylation assays. PstP_c_^D38G^ and PstP_c_^D229G^ were efficiently phosphorylated by both PknA_c_ and PknB_c_ (Figure 3A), whereas faint signal on PstP_c_^R20G^ was observed owing to its partial phosphatase activity. Phosphorylation of PstP_c_ (at 3 µg concentration) was not observed by *in vitro* kinase assay as it completely dephosphorylated PknA_c_ and PknB_c_, making them inactive (heat-inactive PstP_c_ was found to be phosphorylated-data not shown). To confirm that the observed phosphorylation is on PstP_c_-mutants and not on the N-terminally attached His_6_-tag, TEV-protease cleavage of the tag was performed after the kinase assays. Phosphorylation was confirmed to be specifically localized on the cleaved substrate protein (data not shown). Additionally, the R20G, D38G and D229G mutants were also created in full length PstP construct and *p*NPP-hydrolysis assays and phosphorylation reactions were also confirmed using full length PstP and its mutants (data not shown).

### Phosphoamino acid analysis and identification of phosphorylation site(s) of PknA and PknB in PstP_c_^D38G^

Phosphoamino acid analysis by two-dimensional thin layer electrophoresis showed that both PknA_c_ (Figure 3B, upper panel) and PknB_c_ (Figure 3B, lower panel) phosphorylated PstP_c_^D38G^ on Thr residues while no signal was observed on the spots corresponding to pSer and pTyr. For further experiments, PstP_c_^D38G^ was utilized.

The sites of PknA and PknB phosphorylation on PstP_c_^D38G^ were identified through mass-spectrometric analysis by Thermo-Finnagen LTQ electrospray Mass-Spectrometer, using *in vitro* phosphorylated protein. The results showed that four Thr residues were phosphorylated by PknB (Thr^137^, Thr^141^, Thr^174^ and Thr^290^) while PknA phosphorylated PstP_c_^D38G^ on two residues (Thr^137^ and Thr^174^) (Figure 3C, supplementary file 2). Thus, PstP_c_^D38G^ is differentially phosphorylated by PknA and PknB which may have important implications on the activity of PstP.

### Validation of PstP phosphorylation in *E. coli*

**Figure 4:**
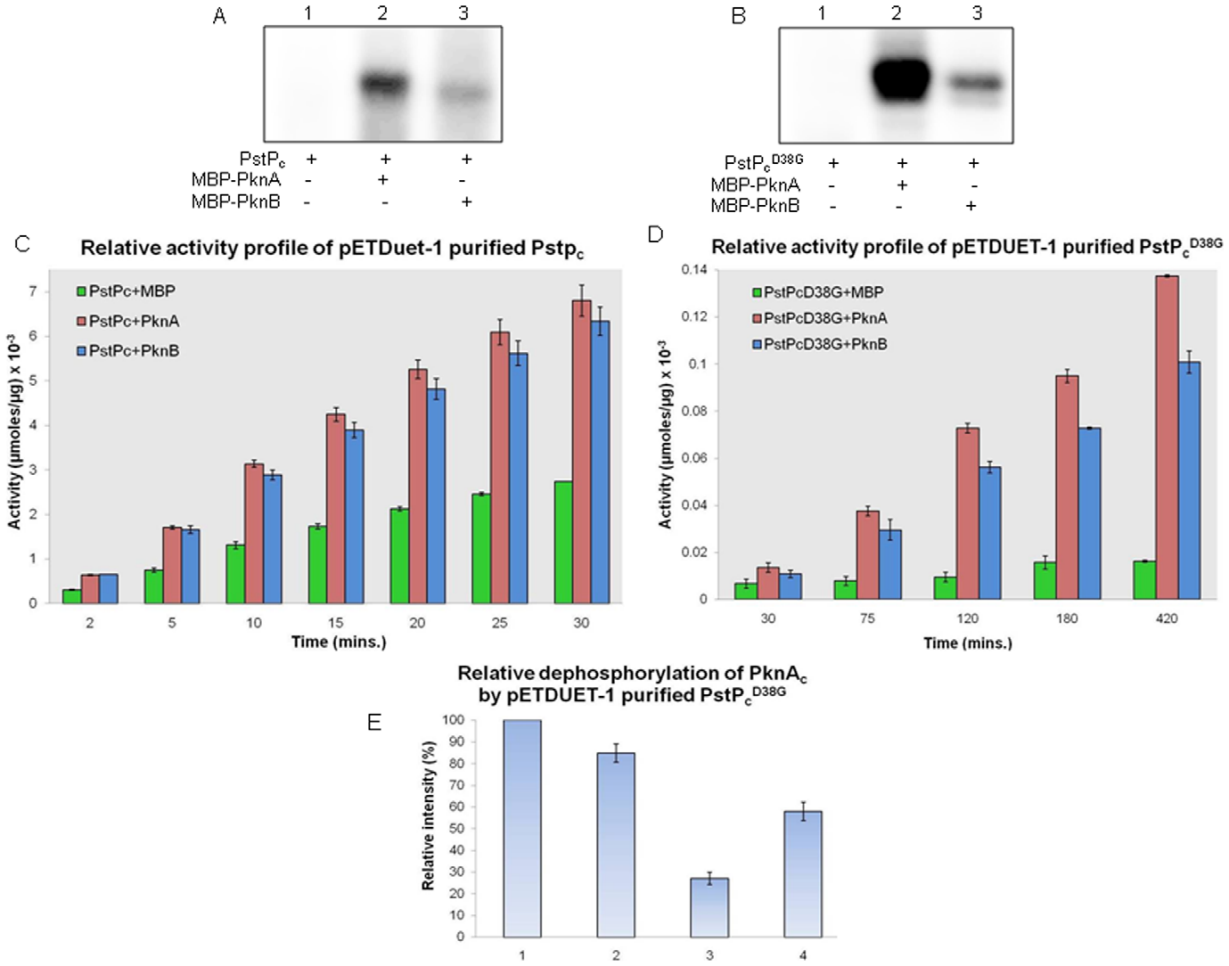
Co-expression analysis of STPKs and PstP_c_/PstP_c_^D38G^. (**A**) Metabolic labeling of PstP_c_: PstP_c_ co-expressed with MBP-PknA (lane 2) or MBP-PknB (lane 3) gets phosphorylated in *E. coli* under native conditions while PstP_c_ co-expressed with MBP alone (lane 1) was not phosphorylated. (**B**) Metabolic labeling of PstP_c_^D38G^: PstP_c_^D38G^ co-expressed with MBP-PknA (lane 2) or MBP-PknB (lane 3) gets phosphorylated in *E. coli* while PstP_c_^D38G^ co-expressed with MBP alone (lane 1) was not phosphorylated. As expected, the intensity of phosphorylation on PstP_c_^D38G^ was comparatively higher than that of PstP_c_. (**C**) Relative activity profile of pETDuet1 purified PstP_c_ and (**D**) PstP_c_^D38G^: *p*NPP assays were performed with PstP_c_ and PstP_c_^D38G^ (1 µg each) purified from pETDuet1 co-expressing MBP or MBP-PknA/PknB. The dephosphorylation potential of phosphorylated PstP_c_ and PstP_c_^D38G^ (co-expressed with either kinase) is higher than that of unphosphorylated protein. For PstP_c_^D38G^, activity was evaluated over long time points due to its low dephosphorylation activity. Activity is calculated as a measure of µmoles of *p*NPP hydrolyzed per µg of protein at a given time. The error bars indicate the SD of three individual experiments. (**E**) Relative dephosphorylation of PknA_c_ by pETDuet-1 purified PstP_c_^D38G^: Autophosphorylated PknA_c_ was incubated for 30 mins with unphosphorylated and phosphorylated PstP_c_^D38G^ and the extent of dephosphorylation was assessed by *in vitro* dephosphorylation assays. The image obtained after autoradiography was analyzed by ImageGauge software (Fuji) and relative intensity of phosphorylation was measured: (1) PknA_c_ alone, (2) PknA_c_+MBP-PstP_c_^D38G^, (3) PknA_c_+PstP_c_^D38G^ phosphorylated by PknA and (4) PknA_c_+PstP_c_^D38G^ phosphorylated by PknB. As shown, the PknA-phosphorylated PstP_c_^D38G^ dephosphorylated the kinase to a greater extent in comparison to the unphosphorylated PstP_c_^D38G^. The error bars represent the SD of the three individual experiments. The corresponding autoradiogram is shown in Figure S4.

To further substantiate our results, the phosphorylation status of PstP_c_ and PstP_c_^D38G^ was examined specifically by PknA and PknB in *E. coli* using a dual expression system. PstP_c_ and PstP_c_^D38G^ were cloned in pETDuet1 expression vector along with either MBP alone or MBP-tagged PknA or PknB. *E. coli* BL21 (DE3) cells transformed with *pETDuet1-PstP_c_/PstP_c_^D38G^-MBP* or *pETDuet1-PstP_c_/PstP_c_^D38G^-MBP-kinase* (kinase, PknA or PknB) were metabolically labelled with [^32^P]orthophosphoric acid. Phosphorylation of PstP_c_ and PstP_c_^D38G^ could only be detected when PknA or PknB were co-expressed (Figures 4A and 4B), suggesting the phosphorylation of phosphatase by both the kinases in native conditions in *E. coli*. Western blot analysis of Ni^2+^-NTA purified samples using rabbit anti-PstP_c_ antibodies confirmed the metabolically labelled protein to be PstP_c_ (data not shown).

### Activity assays of pETDuet1-purified PstP_c_ and PstP_c_^D38G^

The activity profiles of PstP_c_ and PstP_c_^D38G^ co-expressed with and without PknA/PknB, were evaluated. According to the *p*NPP assays, the activity of phosphorylated PstP_c_ (co-expressed with PknA or PknB) was higher than that of unphosphorylated phosphatase (co-expressed with MBP alone) (Figure 4C). The phenomenon was also confirmed by measuring the activity of PstP_c_^D38G^. As already discussed, PstP_c_^D38G^ had retained about 10% of the dephosphorylation activity as a result of which, it was phosphorylated efficiently by kinases. The relative activity of phosphorylated PstP_c_^D38G^ with PknA/PknB and unphosphorylated protein was measured for 420 min. Interestingly, the activity of phosphorylated PstP_c_^D38G^ was remarkably higher than that of unphosphorylated protein, thus the similar profile as that of PstP_c_ was observed (Figure 4D). Also, the activity of PknA phosphorylated phosphatase was even more than the protein phosphorylated by PknB. Noticeably, the increase in phosphatase activity after phosphorylation may also account for the observed increase in the activity of PstP_c_^D229G^ in the time-dependent dephosphorylation assays (Figure 2A).

The dephosphorylation of *in vitro* autophosphorylated PknA_c_ was assessed by PstP_c_^D38G^+MBP, PstP_c_^D38G^+MBP-PknA and PstP_c_^D38G^+MBP-PknB. As expected, due to higher activity of phosphorylated PstP_c_^D38G^, intensity of phosphorylation on PknA_c_ was low as compared to the reaction containing unphosphorylated PstP_c_^D38G^+MBP (Figures 4E and S4). Also, since PknA-phosphorylated PstP_c_^D38G^ was more active than PknB-phosphorylated PstP_c_^D38G^ (Figure 4D), the extent of dephosphorylation was more in lane 3 as compared to lane 4.

### Auto-dephosphorylation of PstP_c_

**Figure 5:**
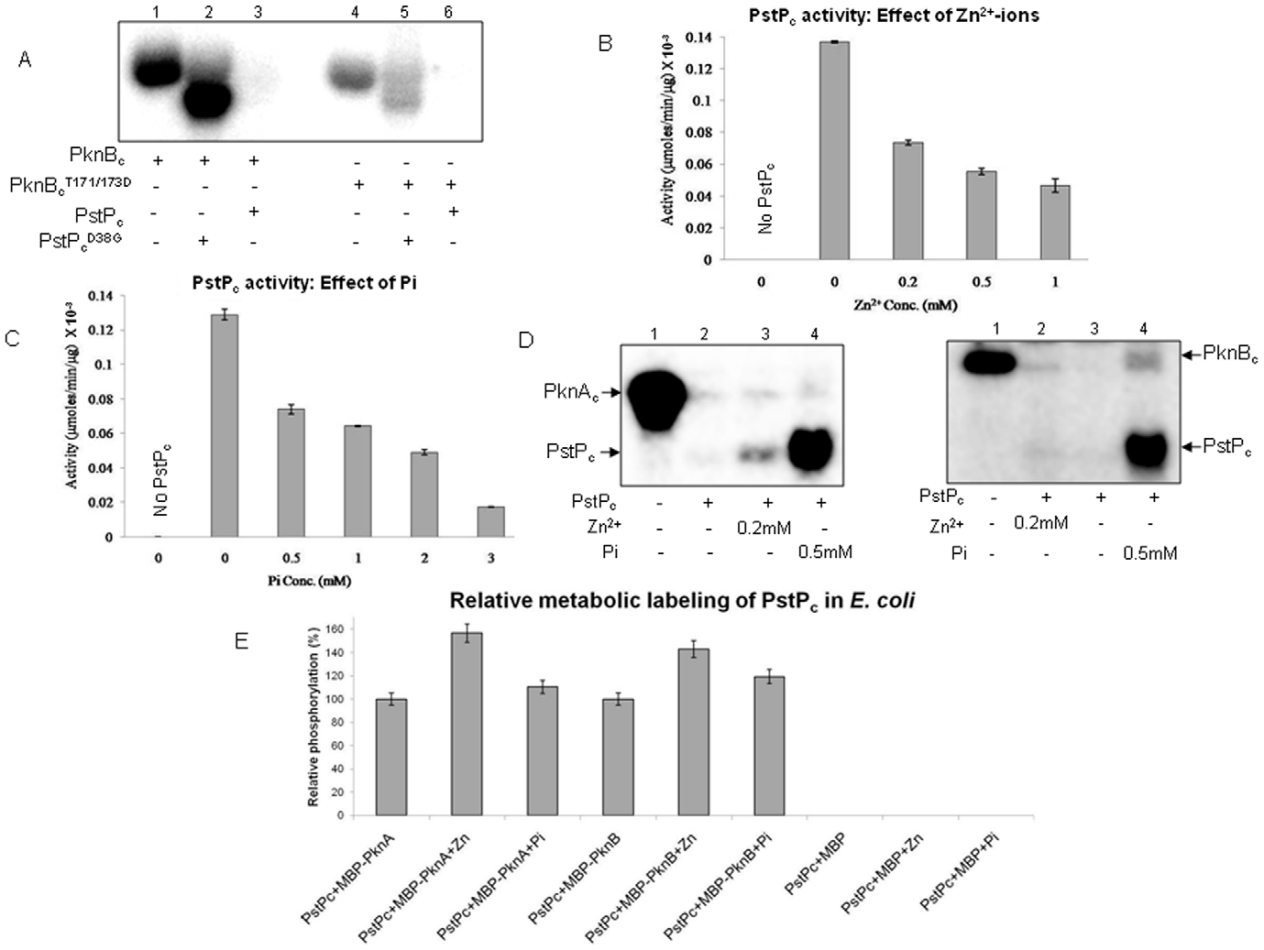
Factors affecting PstP activity. (**A**) Auto-dephosphorylation of PstP_c_: Autoradiogram showing phosphorylation by PknB_c_. PstP_c_ and PstP_c_^D38G^ (3 µg each) were used for *in vitro* phosphorylation assay by PknB_c_ and PknB_c_^T171/173D^ (2 µg each). Since PknB_c_^T171/173D^ cannot be dephosphorylated by PstP_c_, lack of signal signifies auto-dephosphorylation of phosphatase. PstP_c_^D38G^ was used as positive control to show that PknB_c_^T171/173D^ is active. Regulation of PstP_c_ activity: *p*NPP assay showing the effect on activity of PstP_c_ (1 µg) by (**B**) Zn^2+^ and (**C**) Pi. *p*NPP assay was carried out for 30 mins and activity was calculated as a measure of µmoles of *p*NPP hydrolyzed per min per µg of protein. The error bars show SD of three independent experiments. (**D**) Phosphorylation of PstP_c_: Autoradiogram showing the phosphorylation of PstP_c_ (1 µg) by GST-PknA_c_ (left panel) and GST-PknB_c_ (right panel) in presence of 0.2 mM Zn^2+^ and 0.5 mM Pi. Since His_6_-tagged STPKs were not resolved properly from PstP_c_ on SDS-PAGE (Figure S5), the assay was also performed with GST-tagged kinases having higher molecular weights. (**E**) Metabolic labeling of PstP_c_ by PknA and PknB in *E. coli* in presence of Zn^2+^ and Pi: Phosphorylation level of PstP_c_ was observed to be increased when Zn^2+^ (4 mM) and Pi (2 mM) were added during the culture conditions and subsequent processing steps. The autoradiograms obtained after SDS-PAGE were analyzed by ImageGauge software and intensity of the band corresponding to PstP_c_ phosphorylation without any added factor was taken as 100%. Relative phosphorylation is depicted in the bar graph.

Next, we tried to understand whether the inability of PstP_c_ to be effectively phosphorylated was due to its dephosphorylation activity on the kinases resulting in their inactivation or it was due to auto-dephosphorylation. Consequently, phosphomimetic mutants of PknB_c_ were generated for the Thr residues of activation loop in catalytic domain [12], forming PknB_c_^T171/173D^ which cannot be dephosphorylated by PstP_c_ on Thr^171^ and Thr^173^. As reported by Boitel *et* al., PknB does not lose phosphorylation signals after mutagenesis of Thr^171^ and Thr^173^. Through a series of careful analysis of single and double mutants of PknB, it has been shown that PknB can be additionally phosphorylated on Ser^166^ and/or Ser^169^ residues [12]. Thus, we utilized PknB_c_ and PknB_c_^T171/173D^, that were autophosphorylated in an *in vitro* kinase assay using [γ-^32^P]ATP, before incubation with PstP_c_. Phosphorylation of PstP_c_ was still not observed with constitutively active PknB_c_^T171/173D^, as confirmed by phosphotransfer observed on PstP_c_^D38G^ (Figure 5A). This suggests that PstP_c_ can dephosphorylate itself. Additionally, PknB_c_^T171/173D^ was completely dephosphorylated in presence of PstP_c_, suggesting that PstP could also dephosphorylate the surplus sites Ser^166^/Ser^169^.

### Identification of the factors affecting the activity of PstP

The phosphorylation of PstP suggested that additional factors may be involved in the cellular milieu that can regulate and control the phosphatase activity, preceding its phosphorylation. In general, phosphatases are known to be affected by a number of factors like metal-cations, Pi, creatine phosphate (CP) and ATP/ADP ratio. PstP_c_ activity assay was carried out in the presence of selected factors. Interestingly, activity of PstP_c_ was reduced in the presence of Zn^2+^ and Pi, as assessed by *p*NPP assay. Reduction of almost 50% activity was observed at 0.2 mM Zn^2+^ (Figure 5B) and 0.5 mM Pi (Figure 5C). Maximum inhibition of PstP_c_ was observed at 1 mM Zn^2+^ and 4 mM Pi. Inhibition by Zn^2+^ at >1 mM was not calculable due to protein precipitation in the reaction mixture.

### Phosphorylation of PstP_c_ in the presence of Zn^2+^ and Pi

The inhibition of PstP_c_ in the presence of Zn^2+^ and Pi provided a condition that could favour the phosphorylation of PstP_c_ by STPKs. PstP_c_ was indeed phosphorylated by PknA_c_ and PknB_c_ in presence of 0.2 mM Zn^2+^ or 0.5 mM Pi (Figure 5D), under *in vitro* conditions. Since the phosphorylated bands of His_6_-tagged PknA_c_/PknB_c_ and PstP_c_ were not able to resolve on SDS-PAGE (Figure S5), the assay was performed with GST-tagged kinases and similar results were obtained. To further assess the effects of Zn^2+^ and Pi, metabolic labeling of PstP_c_ by co-expressed kinases PknA and PknB was performed in *E. coli* in the presence of Zn^2+^ (4 mM) and Pi (2 mM) (Figure 5E). Phosphorylation of PstP_c_ was indeed enhanced in the presence of Zn^2+^ by ∼40%-50%. The enhancement in phosphorylation in the presence of Pi was not as prominent (∼10%–20%), possibly due to competition of phosphate ions with [^32^P]orthophosphoric acid. Nevertheless, as a proof of principle, Zn^2+^ and Pi were identified as the novel regulators which can inhibit the activity of PstP_c_ and facilitate its phosphorylation.

## Discussion

The coordinated regulation of Ser/Thr protein kinases and phosphatases is essential for maintaining the appropriate equilibrium of protein phosphorylation. Membrane associated kinases and phosphatases are known or hypothesized to be regulated by external stimulus. It is of great relevance to decipher the regulatory mechanisms especially in the systems like *M. tuberculosis* where one Ser/Thr phosphatase PstP is accountable for the effects caused by 11 STPKs. In general, the processes involved in regulating the phosphatases include some external signals, variation in pH [27], cellular concentrations of ATP, ADP, Pi (or their ratios) [28], [29], cytosolic cations like Mn^2+^, Zn^2+^, Mg^2+^, Ca^2+^ [13], [27], [29]–[31] and post-translation modifications (phosphorylation, methylation) [28], [30], [32]–[39]. Present study demonstrates an example of PknA and PknB mediated regulation of PstP through inter-dependent phosphorylation-dephosphorylation reactions. Regulation of phosphatases by phosphorylation is a critical step for cell signaling pathways. It is also associated with feedback phenomena in case where phosphatases are phosphorylated by the kinases that are in turn dephosphorylated by the same phosphatase. Certain examples illustrate the phosphorylation of PP2C phosphatases such as rat Mg^2+^-dependent protein phosphatase α (MPPα) by casein kinase II [39], Soybean kinase associated protein phosphatase (Soybean KAPP) [37], *Oryza sativa* KAPP [40], but these have not been detailed in terms of feedback regulation.

PstP has conserved domain architecture of PP2C-phosphatases (PPM family). PPM family phosphatases play an imperative role in a number of systems described earlier [41]–[48]. Except a few PP2C-phosphatases like Human PP2Cα [49] and Arabidopsis KAPP [50], not much is known about other members of this family. For PstP, we have previously shown that PknA and PknB are the targets for dephosphorylation by PstP and detailed the basic biochemical requirements of this enzyme along with its membrane localization [13]. In a later study, Pullen *et* al. resolved the crystal structure of PstP catalytic domain and described the most important features of this molecule having characteristic PP2C-fold along with three-metal binding centers that associate with Mn^2+^ [18]. The discovery of third-metal centre was a unique feature of PstP as other PP2C phosphatases were found to have two metal-binding centres. In the recent studies, the PP2C-phosphatases of *Streptococcus agalactiae* and *Thermosynechococcus elongatus* have been shown to have a similar third-metal binding centre [51], [52]. The third metal ion center in PstP is proposed to be involved in structural perturbations leading to altered phosphoprotein recognition profiles.

In this study, three conserved residues were selected for generation of site-directed mutants in PstP_c_, on the basis of similarity with Human phosphatase PP2Cα [18]. Arg^20^ (PP2Cα Arg^33^) is responsible for hydrolysis of phosphate moiety from pSer/pThr residues in target proteins. Asp^38^ (PP2Cα Asp^60^) and Asp^229^ (PP2Cα Asp^282^) constitute a part of Mn^2+^-metal centers and coordinate with the two critical Mn^2+^. Mutations of Asp^38^ and Asp^229^ affected the activity of PstP rendering it active to minimal level, though R20G mutant retained about 40% activity. Thus, the residues that are involved in Mn^2+^-ion binding and hydrolysis of phosphate are deciphered to be critical for its activity. Accordingly, the extent of phosphorylation of each mutant was dependent on the remaining dephosphorylation activity, so that PstP_c_^D38G^ and PstP_c_^D229G^ were efficiently phosphorylated by PknA and PknB.

Association with metals is crucial for PP2C phosphatases and any perturbation with inherently associated metals may lead to altered functional profile. The minimum requirement for PstP_c_ activity is the presence of Mn^2+^ [13]. For PP2C-class of phosphatases, divalent ions other than Mn^2+^/Mg^2+^ can inhibit their activity by competitively replacing the Mn^2+^ in the core enzyme structure [27] and Zn^2+^ are the most potent regulators, having comparable ionic radii with that of Mn^2+^. PstP_c_ was partially inactive in the presence of 0.2 mM ZnCl_2_ and displayed lower activity on increasing the Zn^2+^-ion concentration upto 2 mM, as observed by *p*NPP assays. *In vitro* kinase assays with PknA_c_ and PknB_c_ in presence of Zn^2+^ resulted in phosphorylation of PstP_c_. Also, there was increase in phosphorylation of PstP_c_ during metabolic labeling by PknA and PknB in the presence of Zn^2+^ added in the *E. coli* culture. These results indicate that in mycobacterial cell, if cytosolic Zn^2+^ concentration increases, it may inhibit PstP perhaps leading to its phosphorylation. In an elaborative elemental analysis, Wagner *et* al. have reported that during infection, intravacuolar Zn^2+^-ion concentration increases from 0.037 mM to 0.46 mM in macrophages infected with *M. tuberculosis* [53]. Although there is no report of concomitant increase in mycobacterial Zn^2+^-ion concentration, it can only be speculated that if these changes in vacuolar ionic concentrations alter the mycobacterial ionic profile, a condition may develop where the enzymes that respond to Zn^2+^ (like PstP) can be activated or deactivated.

End-product inhibition of enzymes is a well established phenomenon to prevent the accumulation of a particular metabolite. In case of reversible reactions, end-product accumulation can change the direction of the reaction. Similarly, Pi is known to inhibit a number of phosphatases [27], [42], [49] and in present study, PstP_c_ mediated *p*NPP hydrolysis is inhibited by Pi. To confirm that this effect is not limited to *p*NPP, *in vitro* kinase assays and metabolic labeling in *E. coli* showed PstP_c_ to be phosphorylated by PknA and PknB in presence of Pi because of its inhibition. Pi content is indicative of nutrient availability and energy status of the cell. In general, high Pi is associated with energy-starved conditions, when all the ATP is depleted and metabolite homeostasis is in unbalanced state. Such conditions usually arise during late-log and stationary phases in culture conditions.

Metabolic labeling by [^32^P]orthophosphoric acid in the presence of co-expressed STPK (PknA or PknB) in *E. coli* lead to the specific phosphorylation of PstP_c_ and PstP_c_^D38G^. Co-expression in pETDuet-1 has previously been utilized extensively to assess the interaction of mycobacterial STPKs with their cognate substrates in the surrogate host *E. coli* [21], [23]. Such dual-expression systems are increasingly becoming useful for analysis of protein-protein interactions specifically for challenging systems like mycobacteria [54]. Activity assays of the pETDuet-1 purified PstP_c_ and PstP_c_^D38G^ revealed the higher activity of PknA-phosphorylated phosphatase as compared to the PknB-phosphorylated protein. Prominent variations in the activity of phosphorylated and unphosphorylated PstP_c_^D38G^ were observed with phosphorylated protein being proficient to hydrolyze *p*NPP to a greater extent (∼15-fold higher) in contrast to the unphosphorylated protein. The difference in the activities of phosphorylated and unphosphorylated PstP_c_ was not as prominent as that of PstP_c_^D38G^ (∼2–3 fold higher). These differences may be attributed to the fact that PstP_c_ may get auto-dephosphorylated to a greater extent than PstP_c_^D38G^ during expression and purification procedures. Higher activity of phosphorylated phosphatase is suggestive of reverse regulation of signaling cascade emanating from the kinases. In the constitutively active state, STPKs perform their regular functions and phosphorylate the target substrates following the stimulus. This may ultimately lead to the phosphorylation of PstP. The resulting increase in the activity of phosphatase may itself act as a control mechanism for kinases, eventually impeding the continued effect of that particular stimulus. The overall process has to be dynamic due to auto-dephosphorylation of PstP, eventually ceasing the effect of signaling cascade. In the conditions of high Zn^2+^ or high Pi content of the cell, PstP may not be active and will allow the kinase to work at its maximal activity. The proposed phosphorylation of PstP in such conditions may act as a mechanism to overcome the inhibition of PstP, hence balancing the cellular signaling pathways.

## Supporting Information

Figure S1 ***p*** **NPP-assay.** To confirm the authenticity of pNPP assay, increasing amounts of alkaline phosphatase (0-100 ng) was used a positive control and PknB_c_ (0–5 µg) was used as a negative control. The assay was performed for 30 mins at 37°C and the activity is calculated as µmoles of *p*NPP hydrolyzed per min at a given amount of enzyme used. As clearly evident, alkaline phosphatase showed very high activity while no such activity was detected in PknB_c_.
(TIF)

Figure S2 **Effect of mutations on the activity of PstP_c_.** To show that the loss in activity of PstP_c_ was specifically due to mutations of Arg^20^, Asp^38^ and Asp^229^, PstP_c_ was mutagenized on irrelevant residues Thr^5^ and Thr^141^ to Ala and Glu, respectively and *p*NPP hydrolysis was performed for 30 mins at 37°C. Activity of PstP_c_ was taken as 100% and relative activity was calculated. As evident from the bar graph, there were no significant changes in the activity of the mutants PstP_c_^T5A^ and PstP_c_^T141E^ as compared to PstP_c_.
(TIF)

Figure S3 **Time-dependent** ***p*** **NPP-assay.** *p*NPP-hydrolysis was performed in a time-dependent manner for 30 mins using PstP_c_, PstP_c_^R20G^, PstP_c_^D38G^ and PstP_c_^D229G^ variants (2 µg each) at 37°C. Alkaline phosphatase (2 ng) was used a positive control and PknB_c_ (5 µg) was used as a negative control. Activity was calculated as nmoles of *p*NPP hydrolyzed per µg of enzyme used at a given time and depicted in logarithmic scale. Nevertheless, the results are essentially similar as that of time-dependent dephosphorylation of PknB_c_ (Figure 2A).
(TIF)

Figure S4 ***In vitro*** **dephosphorylation activity of pETDuet-1 purified PstP_c_^D38G^.** Autophosphorylated PknA_c_ was incubated with unphosphorylated and phosphorylated PstP_c_^D38G^. As shown in the autoradiogram, the PknA-phosphorylated PstP_c_^D38G^ dephosphorylated the kinase to a greater extent in comparison to the unphosphorylated PstP_c_^D38G^. The image was also analyzed by ImageGauge software and corresponding values are depicted by bar-graph (Figure 4E).
(TIF)

Figure S5 **Phosphorylation of PstP_c_.** Autoradiogram showing the phosphorylation of PstP_c_ (1 µg) by His_6_-tagged STPKs PknA_c_ (upper panel) and PknB_c_ (lower panel) in presence of 0.2 mM Zn^2+^ and 0.5 mM Pi. Due to overlapping molecular weights of PknA_c_ and PknB_c_ with PstP_c_, the bands were not separated properly. Still, the phosphotransfer on PstP_c_ was evident in presence of Zn^2+^ and Pi by both the kinases. The reaction was also performed with GST-tagged STPKs to clearly depict the reaction (Figure 5D).
(TIF)

File S1 **Detailed protocol of sample processing for identification of phosphorylation sites.**
(DOC)
