# Supplementary figures and images for: Phosphorylation of Mycobacterium tuberculosis Ser/Thr Phosphatase by PknA and PknB
Source: PLoS One. 2011 Mar 9;6(3):e17871. doi: 10.1371/journal.pone.0017871 (PMC3052367; doi:10.1371/journal.pone.0017871)

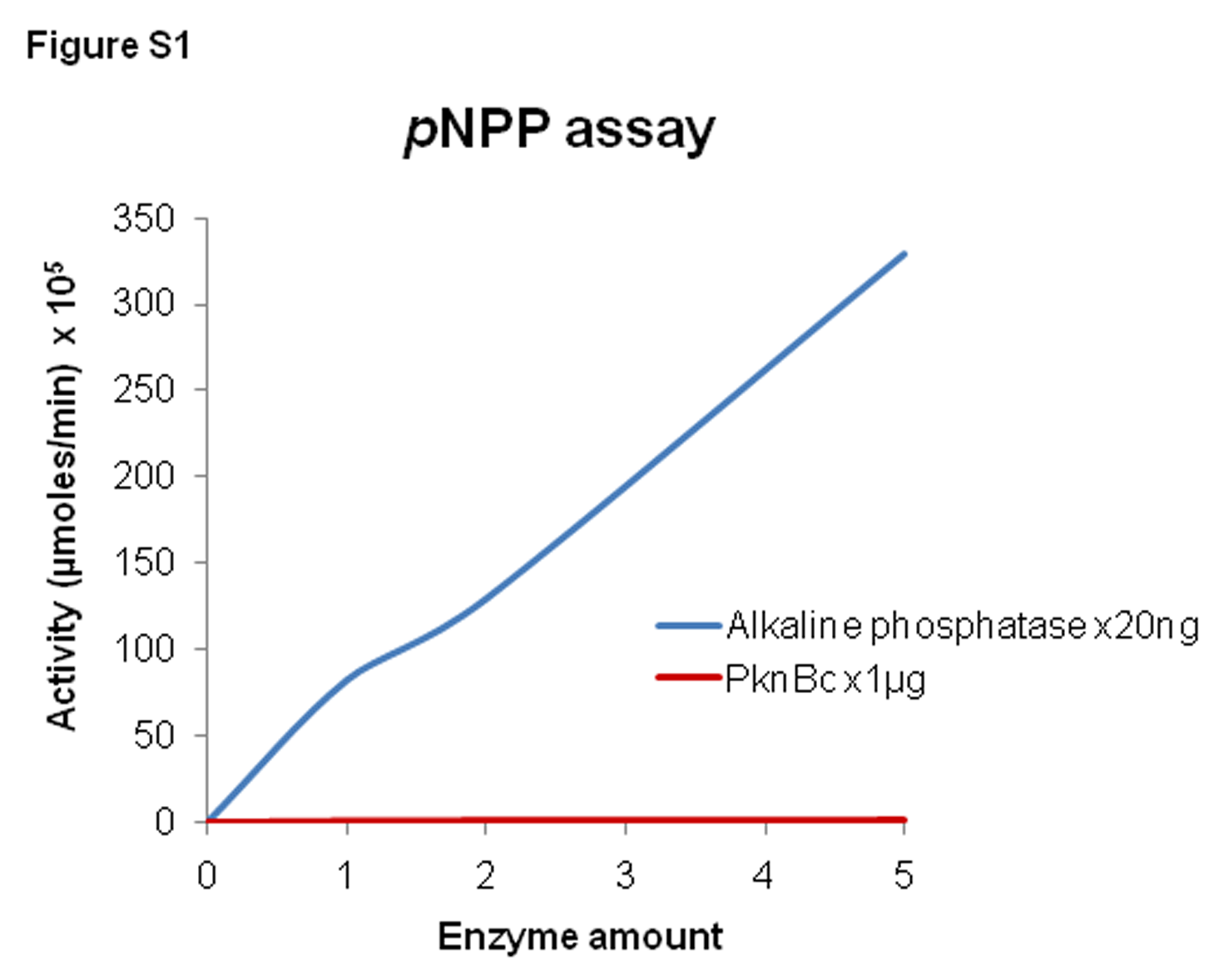

Supplement: Figure S1 — p NPP-assay. To confirm the authenticity of pNPP assay, increasing amounts of alkaline phosphatase (0-100 ng) was used a positive control and PknBc (0–5 µg) was used as a negative control. The assay was performed for 30 mins at 37°C and the activity is calculated as µmoles of pNPP hydrolyzed per min at a given amount of enzyme used. As clearly evident, alkaline phosphatase showed very high activity while no such activity was detected in PknBc. (TIF) [file pone.0017871.s001.tif]

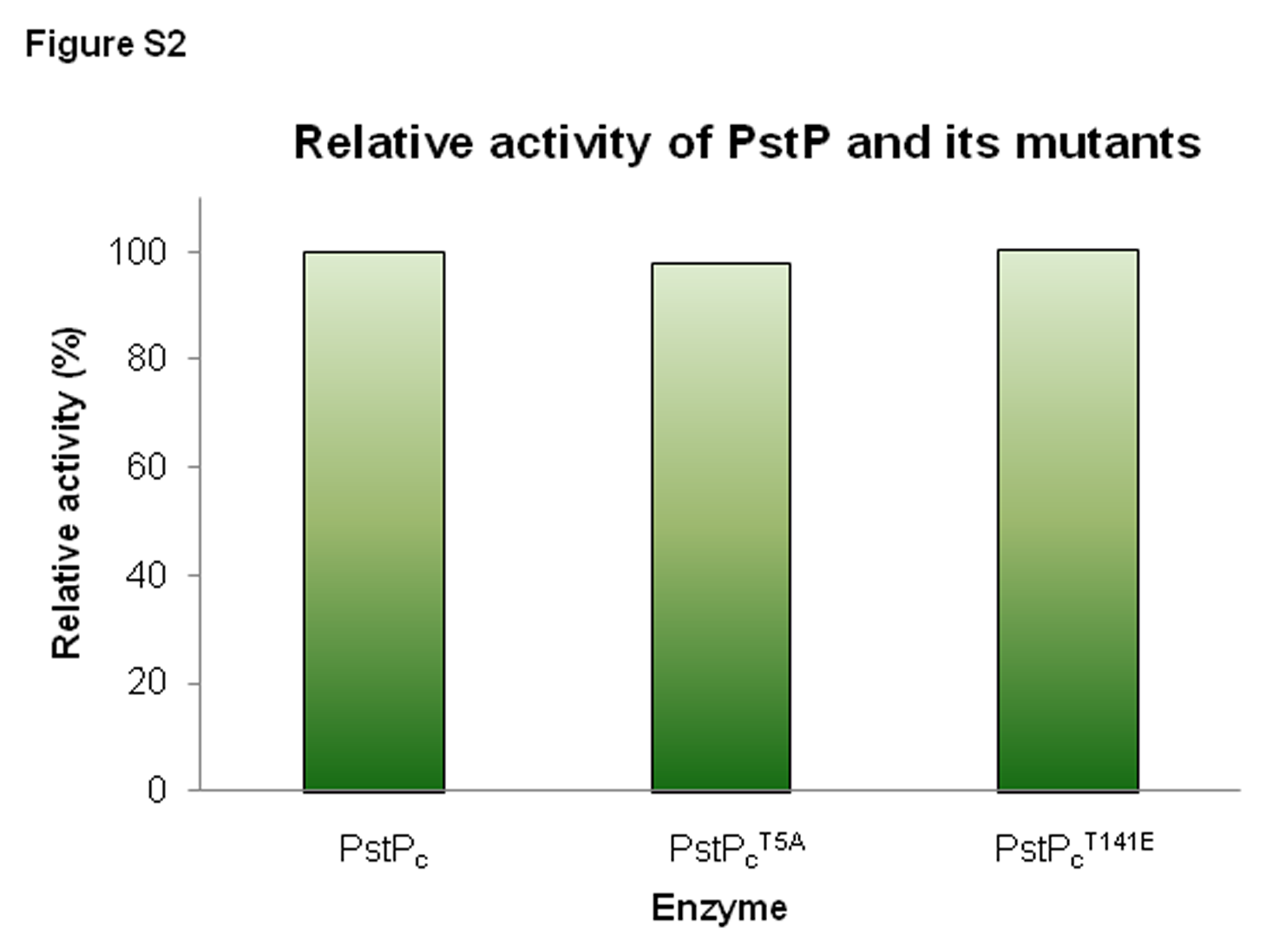

Supplement: Figure S2 — Effect of mutations on the activity of PstPc. To show that the loss in activity of PstPc was specifically due to mutations of Arg20, Asp38 and Asp229, PstPc was mutagenized on irrelevant residues Thr5 and Thr141 to Ala and Glu, respectively and pNPP hydrolysis was performed for 30 mins at 37°C. Activity of PstPc was taken as 100% and relative activity was calculated. As evident from the bar graph, there were no significant changes in the activity of the mutants PstPc T5A and PstPc T141E as compared to PstPc. (TIF) [file pone.0017871.s002.tif]

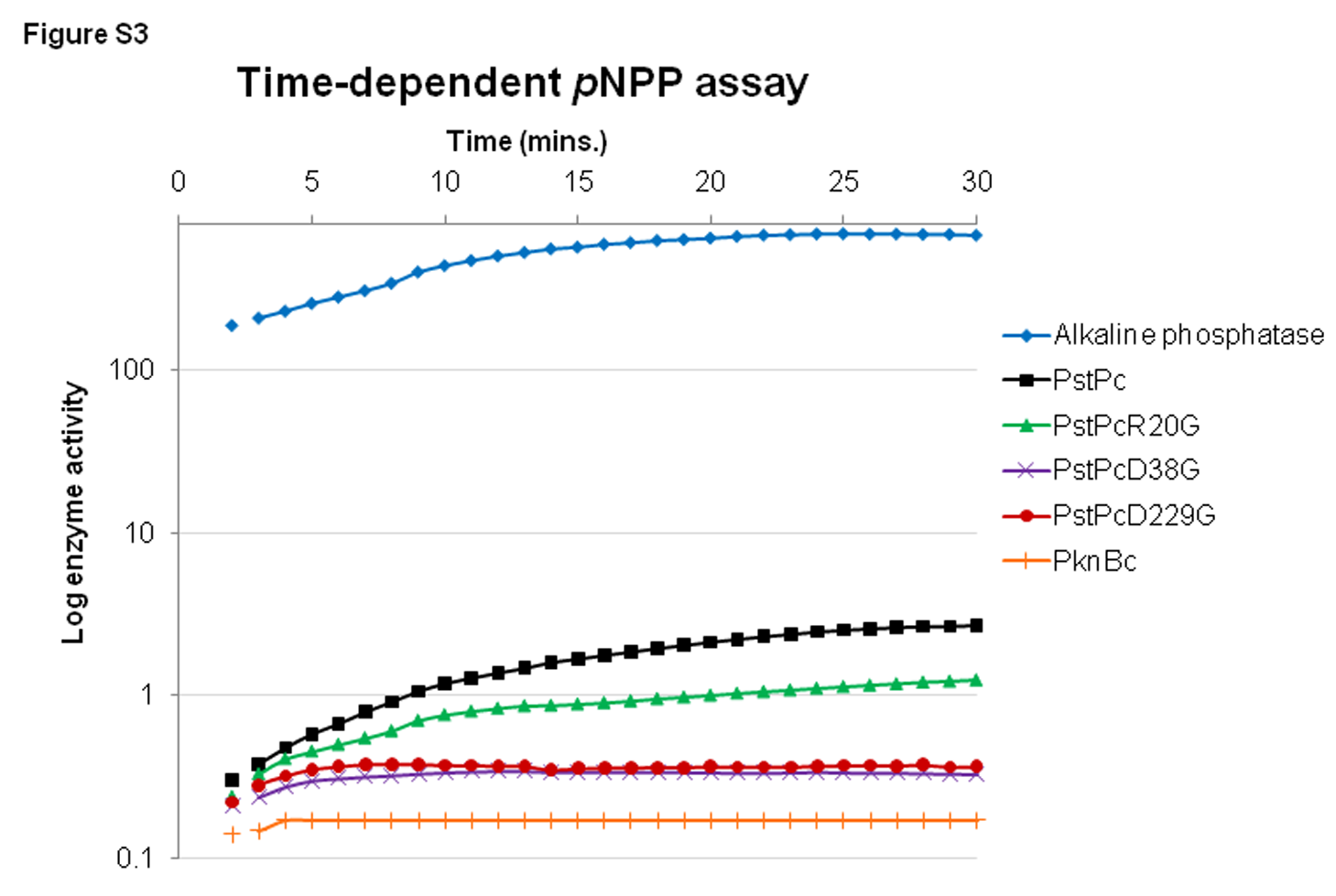

Supplement: Figure S3 — Time-dependent p NPP-assay. pNPP-hydrolysis was performed in a time-dependent manner for 30 mins using PstPc, PstPc R20G, PstPc D38G and PstPc D229G variants (2 µg each) at 37°C. Alkaline phosphatase (2 ng) was used a positive control and PknBc (5 µg) was used as a negative control. Activity was calculated as nmoles of pNPP hydrolyzed per µg of enzyme used at a given time and depicted in logarithmic scale. Nevertheless, the results are essentially similar as that of time-dependent dephosphorylation of PknBc (Figure 2A). (TIF) [file pone.0017871.s003.tif]

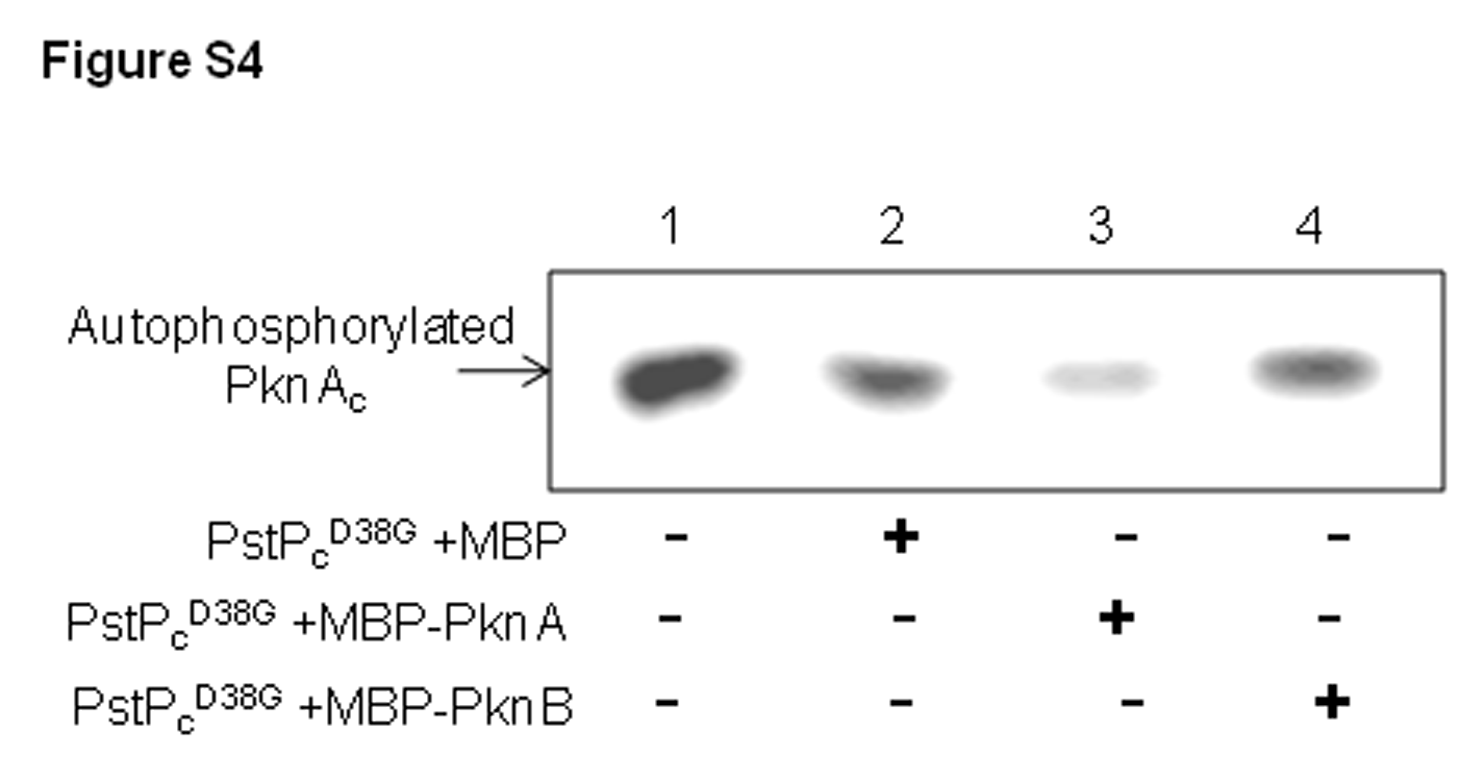

Supplement: Figure S4 — In vitro dephosphorylation activity of pETDuet-1 purified PstPcD38G. Autophosphorylated PknAc was incubated with unphosphorylated and phosphorylated PstPc D38G. As shown in the autoradiogram, the PknA-phosphorylated PstPc D38G dephosphorylated the kinase to a greater extent in comparison to the unphosphorylated PstPc D38G. The image was also analyzed by ImageGauge software and corresponding values are depicted by bar-graph (Figure 4E). (TIF) [file pone.0017871.s004.tif]

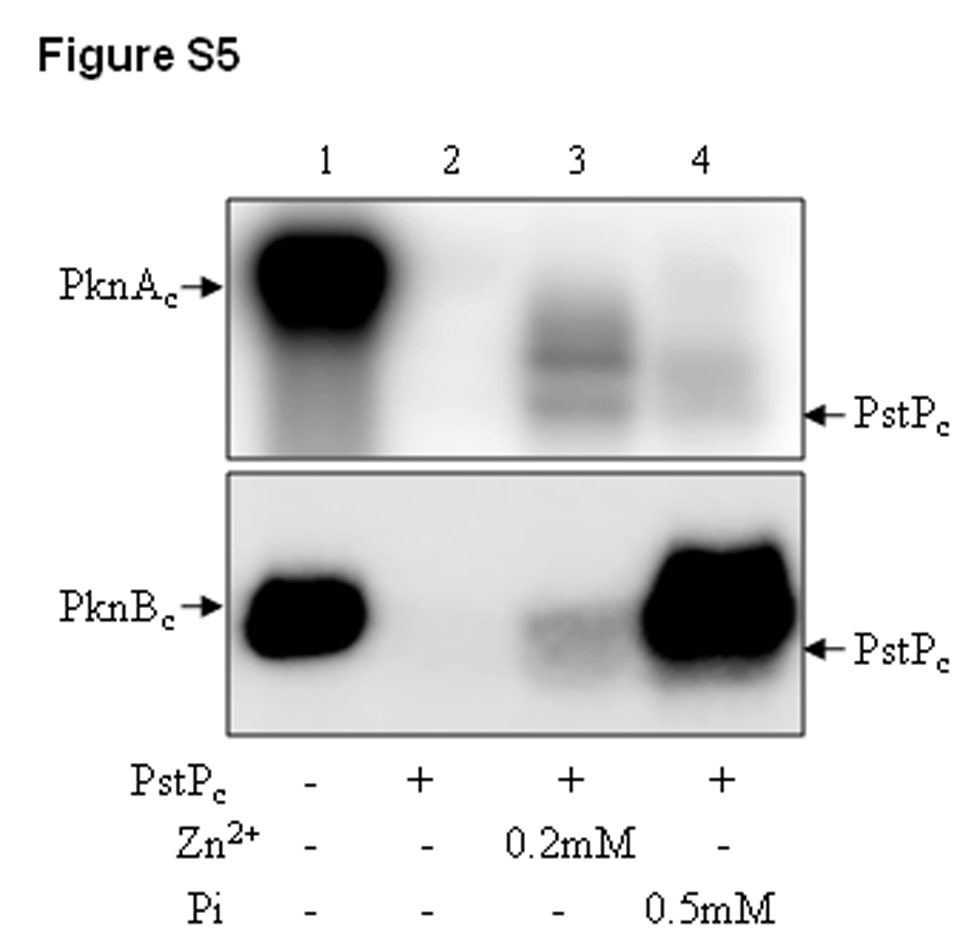

Supplement: Figure S5 — Phosphorylation of PstPc. Autoradiogram showing the phosphorylation of PstPc (1 µg) by His6-tagged STPKs PknAc (upper panel) and PknBc (lower panel) in presence of 0.2 mM Zn2+ and 0.5 mM Pi. Due to overlapping molecular weights of PknAc and PknBc with PstPc, the bands were not separated properly. Still, the phosphotransfer on PstPc was evident in presence of Zn2+ and Pi by both the kinases. The reaction was also performed with GST-tagged STPKs to clearly depict the reaction (Figure 5D). (TIF) [file pone.0017871.s005.tif]
