# Supplementary material for: Phosphorylation of Mycobacterium tuberculosis Ser/Thr Phosphatase by PknA and PknB
Source: PLoS One. 2011 Mar 9;6(3):e17871. doi: 10.1371/journal.pone.0017871 (PMC3052367; doi:10.1371/journal.pone.0017871)
Supplement: File S1 — Detailed protocol of sample processing for identification of phosphorylation sites. (DOC) [file pone.0017871.s006.doc]

**Detailed protocol of sample processing for identification of phosphorylation sites:**

Bands of interest were digested with sequencing grade trypsin (Promega) at a concentration of 12.5ng/µl in 100mM ammonium bicarbonate at 37°C overnight. The peptides were extracted with 100mM ammonium bicarbonate and acetonitrile followed by lyophilization. The sample was resuspended in 5% acetonitrile and 5% formic acid before it was directly injected into the LC/MS system comprising a micro-autosampler, Suvery HPLC pump and a ProteomeX (LTQ) mass spectrometer (Thermo Finnigan, San Jose, CA). The LC-system featured an in-house packed reversed phase column using C18 Magic (3mm, 200Å; Michrom Bioresource) packing material and PicoTip Emitters (New Objective, Woburn, MA). The peptides were eluted with a 30 min linear gradient and the six most abundant species selected for automated fragmentation. If the fragmentation of a MS spectra resulted in an immediate neutral loss (NL) mimicking the mass of an M/Z value of a phosphate group in the MS2, then an MS3 fragmentation was performed on the NL MS2 peak.

Mass spectrometric data was searched against a non-redundant *M. tuberculosis* database using the protein identification software Mascot (v2.2.04, Matrix Sciences, London, UK). Search criteria included tryptic peptide specificity with one missed cleavage, Carbamidomethyl (C) as a fixed modification, Deamidated (NQ), Gln->pyro-Glu (N-term Q), Phospho (STY) and Oxidation (M) as variable modifications for MS2 spectra. MS3 spectra (triggered by the NL in the MS2) were search with the following criteria; tryptic peptide specificity with one missed cleavage, Carbamidomethyl (C) as a fixed modification, Deamidated (NQ), Gln->pyro-Glu (N-term Q), Dehydrated (ST), Phospho (Y) and Dethiomethyl (M) as variable modifications. A 1.5Da peptide tolerance for the MS and a 0.8Da peptide tolerance for MS/MS spectra were implemented along with a mascot ion cut off score of 30. Spectra sequences identified by mascot to have a phosphorylation were validated manually to ensure proper identification of the amino acid location of the modification site and access overall quality of the data.

**Sample 1: PstP_c_^D38G^ without kinase (Control)**

Match to: **gi|1552573** Score: **1529**

**POSSIBLE SERINE/THREONINE PHOSPHATASE PPP [Mycobacterium tuberculosis H37Rv]**

Found in search of 25149

Job #50149 PID 931838

Variable modifications: Carbamidomethyl (C),Deamidated (NQ),Gln->pyro-Glu (N-term Q),Oxidation (M),Phospho (ST),Phospho (Y)

Cleavage by Trypsin: cuts C-term side of KR unless next residue is P

Sequence Coverage: **51%**

Matched peptides shown in **Bold Red,** phosphorylated residues in **Green.**

**1** MAR**VTLVLR**Y AARSDRGLVR **ANNEDSVYAG ARLLALADGM GGHAAGEVAS**

**51 QLVIAALAHL DDDEPGGDLL AKLDAAVRAG NSAIAAQVEM EPDLEGMGTT**

**101 LTAILFAGNR LGLVHIGDSR GYLLRDGELT QITKDDTFVQ TLVDEGRITP**

**151 EEAHSHPQR**S LIMR**ALTGHE VEPTLTMR**EA RAGDR**YLLCS DGLSDPVSDE**

**201 TILEALQIPE VAESAHRLIE LALRGGGPDN VTVVVADVVD YDYGQTQPIL**

**251 AGAVSGDDDQ LTLPNTAAGR ASAISQR**KEI VK**RVPPQADT FSRPR**WSGRR

**301** LAFVVALVTV LMTAGLLIGR AIIRSNYYVA DYAGSVSIMR GIQGSLLGMS

**351** LHQPYLMGCL SPRNELSQIS YGQSGGPLDC HLMKLEDLRP PERAQVRAGL

**401** PAGTLDDAIG QLRELAANSL LPPCPAPRAT SPPGRPAPPT TSETTEPNVT

**451** SSPASPSPTT SAPAPTGTTP AIPTSASPAA PASPPTPWPV TSSPTMAALP

**501** PPPPQPGIDC RAAA

**Sample 2: PstP_c_^D38G^ phosphorylated by PknB_c_**

Match to: **gi|1552573** Score: **1611**

**POSSIBLE SERINE/THREONINE PHOSPHATASE PPP [Mycobacterium tuberculosis H37Rv]**

Found in search of 25144

Job #50150 PID 9780

Variable modifications: Carbamidomethyl (C),Deamidated (NQ),Gln->pyro-Glu (N-term Q),Oxidation (M),Phospho (ST),Phospho (Y)

Cleavage by Trypsin: cuts C-term side of KR unless next residue is P

Sequence Coverage: **28%**

Matched peptides shown in **Bold Red,** phosphorylated residues in **Green.**

**1** MARVTLVLRY AARSDRGLVR **ANNEDSVYAG AR**LLALADGM GGHAAGEVAS

**51** QLVIAALAHL DDDEPGGDLL AKLDAAVR**AG NSAIAAQVEM EPDLEGMGTT**

**101 LTAILFAGNR LGLVHIGDSR GYLLRDGELT QITKDDTFVQ TLVDEGR**ITP

**151** EEAHSHPQRS LIMR**ALTGHE VEPTLTMR**EA RAGDR**YLLCS DGLSDPVSDE**

**201 TILEALQIPE VAESAHRLIE LALR**GGGPDN VTVVVADVVD YDYGQTQPIL

**251** AGAVSGDDDQ LTLPNTAAGR ASAISQRKEI VK**RVPPQADT FSRPR**WSGRR

**301** LAFVVALVTV LMTAGLLIGR AIIRSNYYVA DYAGSVSIMR GIQGSLLGMS

**351** LHQPYLMGCL SPRNELSQIS YGQSGGPLDC HLMKLEDLRP PERAQVRAGL

**401** PAGTLDDAIG QLRELAANSL LPPCPAPRAT SPPGRPAPPT TSETTEPNVT

**451** SSPASPSPTT SAPAPTGTTP AIPTSASPAA PASPPTPWPV TSSPTMAALP

**501** PPPPQPGIDC RAAA

**Sample 3: PstP_c_^D38G^ phosphorylated by PknA_c_**

Match to: **gi|1552573** Score: **2463**

**POSSIBLE SERINE/THREONINE PHOSPHATASE PPP [Mycobacterium tuberculosis H37Rv]**

Found in search of 25152

Job #50148 PID 20813

Variable modifications: Carbamidomethyl (C),Deamidated (NQ),Gln->pyro-Glu (N-term Q),Oxidation (M),Phospho (ST),Phospho (Y)

Cleavage by Trypsin: cuts C-term side of KR unless next residue is P

Sequence Coverage: **39%**

Matched peptides shown in **Bold Red,** phosphorylated residues in **Green.**

**1** MAR**VTLVLR**Y AARSDRGLVR **ANNEDSVYAG AR**LLALADGM GGHAAGEVAS

**51** QLVIAALAHL DDDEPGGDLL AKLDAAVR**AG NSAIAAQVEM EPDLEGMGTT**

**101 LTAILFAGNR LGLVHIGDSR GYLLRDGELT QITKDDTFVQ TLVDEGR**ITP

**151** EEAHSHPQR**S LIMRALTGHE VEPTLTMR**EA RAGDR**YLLCS DGLSDPVSDE**

**201 TILEALQIPE VAESAHRLIE LALRGGGPDN VTVVVADVVD YDYGQTQPIL**

**251 AGAVSGDDDQ LTLPNTAAGR** ASAISQRKEI VK**RVPPQADT FSRPR**WSGRR

**301** LAFVVALVTV LMTAGLLIGR AIIRSNYYVA DYAGSVSIMR GIQGSLLGMS

**351** LHQPYLMGCL SPRNELSQIS YGQSGGPLDC HLMKLEDLRP PERAQVRAGL

**401** PAGTLDDAIG QLRELAANSL LPPCPAPRAT SPPGRPAPPT TSETTEPNVT

**451** SSPASPSPTT SAPAPTGTTP AIPTSASPAA PASPPTPWPV TSSPTMAALP

**501** PPPPQPGIDC RAAA
